# Early economic evaluation of magnetic resonance imaging for prostate cancer detection in primary care

**DOI:** 10.1002/bco2.409

**Published:** 2024-07-10

**Authors:** Samuel William David Merriel, Peter Buttle, Sarah J. Price, Nick Burns‐Cox, Fiona M. Walter, William Hamilton, Anne E. Spencer

**Affiliations:** ^1^ University of Manchester Manchester UK; ^2^ Patient & Public Involvement Swindon United Kingdom; ^3^ University of Exeter Exeter UK; ^4^ Somerset NHS Foundation Trust Taunton UK; ^5^ Queen Mary University London London UK

**Keywords:** bpMRI, diagnosis, early economic evaluation, mpMRI, primary care, Prostate cancer

## Abstract

**Objectives:**

To explore the potential impacts of incorporating prebiopsy magnetic resonance imaging into primary care as a triage test within the prostate cancer diagnostic pathway.

**Subjects and methods:**

Decision analytic modelling with decision trees was utilised for this early economic evaluation. A conceptual model was developed reflecting the common primary care routes to diagnosis for prostate cancer: opportunistic, asymptomatic prostate‐specific antigen (PSA) screening or symptomatic presentation. The use of multiparametric MRI (mpMRI) or biparametric MRI (bpMRI) as a primary care triage test following an elevated PSA result was evaluated. A health system perspective was adopted with a time horizon of 12 months. Health effects were expressed in terms of utilities drawn from the literature. The primary outcome was prostate cancer diagnosis. Evidence used to inform the model was drawn from published primary studies, systematic reviews, and secondary analyses of primary and secondary care datasets.

**Results:**

Base case analysis showed that the PSA pathway was dominated by both mpMRI‐ and bpMRI‐based pathways for patients undergoing opportunistic screening and symptomatic assessment. bpMRI pathways had greater improvement in cost and utility than mpMRI pathways in both clinical scenarios. Significantly more MRI scans would be performed using the modelled approach (66 626 scans vs. 37 456 scans per 100 000 patients per annum), with fewer subsequent urgent suspected cancer referrals for both mpMRI (38% reduction for screening and symptomatic patients) and bpMRI (72% reduction for screening; 71% for symptomatic) pathways, and a small increase in number of missed cancer diagnoses. Deterministic sensitivity analyses, varying each parameter to its upper and lower 95% confidence intervals, showed no significant change in the dominance of the MRI‐based prostate cancer diagnostic pathways.

**Conclusion:**

Using prostate MRI as a second‐level triage test for suspected prostate cancer in primary care could reduce health service costs without a detrimental effect on patient utility.

## 1 INTRODUCTION

**FIGURE 1:**
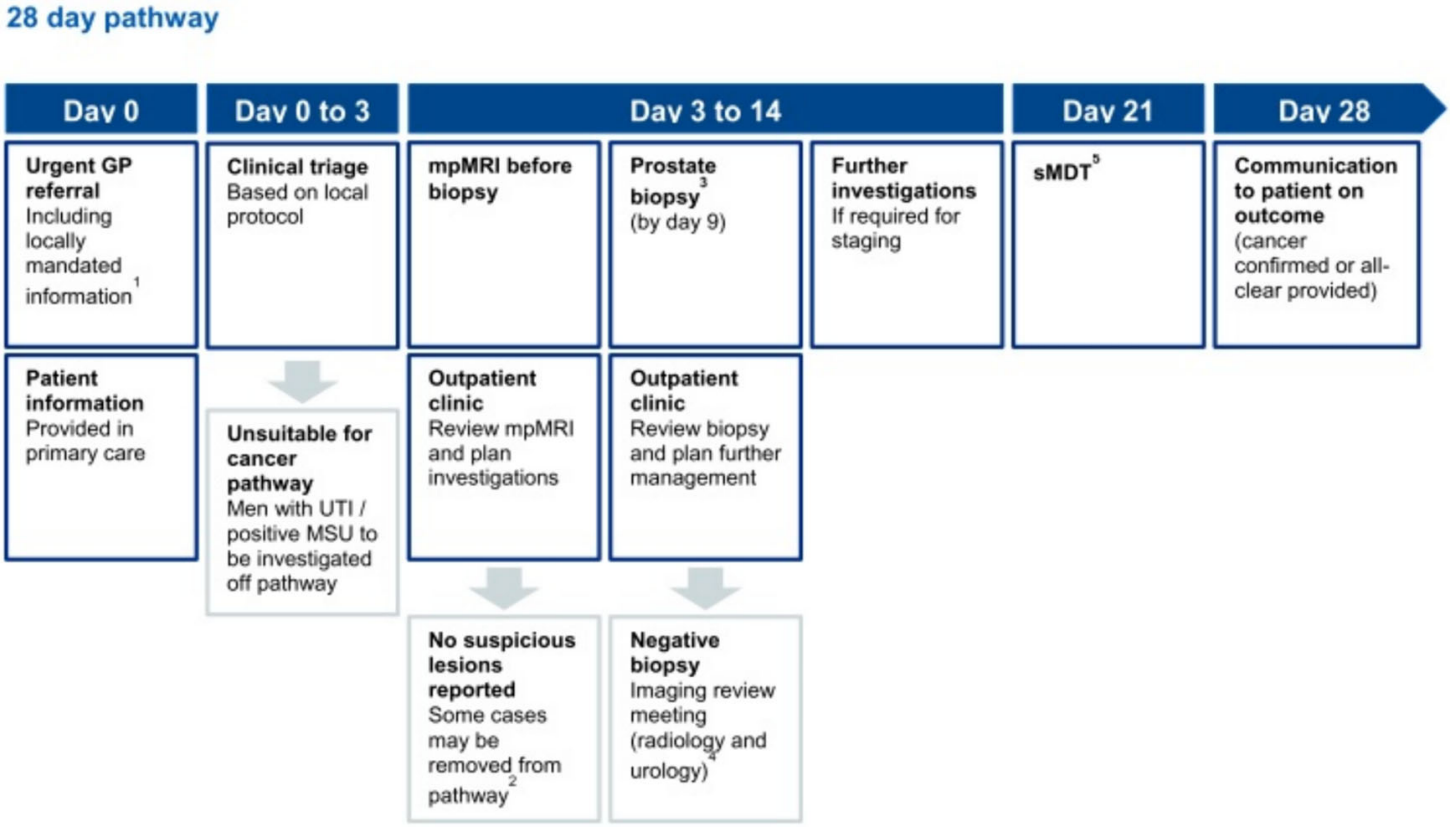
Recommended 28‐day pathway from ‘*Implementing a timed prostate cancer diagnostic pathway [3]*’.

Integration of prostate magnetic resonance imaging (MRI) into existing prostate cancer diagnostic pathways in the NHS has been encouraged since 2017 following publication of the PROMIS^1^ and PRECISION^2^ trials. A 2018 report titled ‘*Implementing a timed prostate cancer diagnostic pathway*’ outlined the case for prebiopsy prostate MRI, based on NHS vanguard pathways implemented in London and Manchester (see Figure 1).^3^ National Institute for Health and Care Excellence (NICE) guidance was updated in 2019 to recommend this approach for the diagnosis of prostate cancer.^4^

**FIGURE 2:**
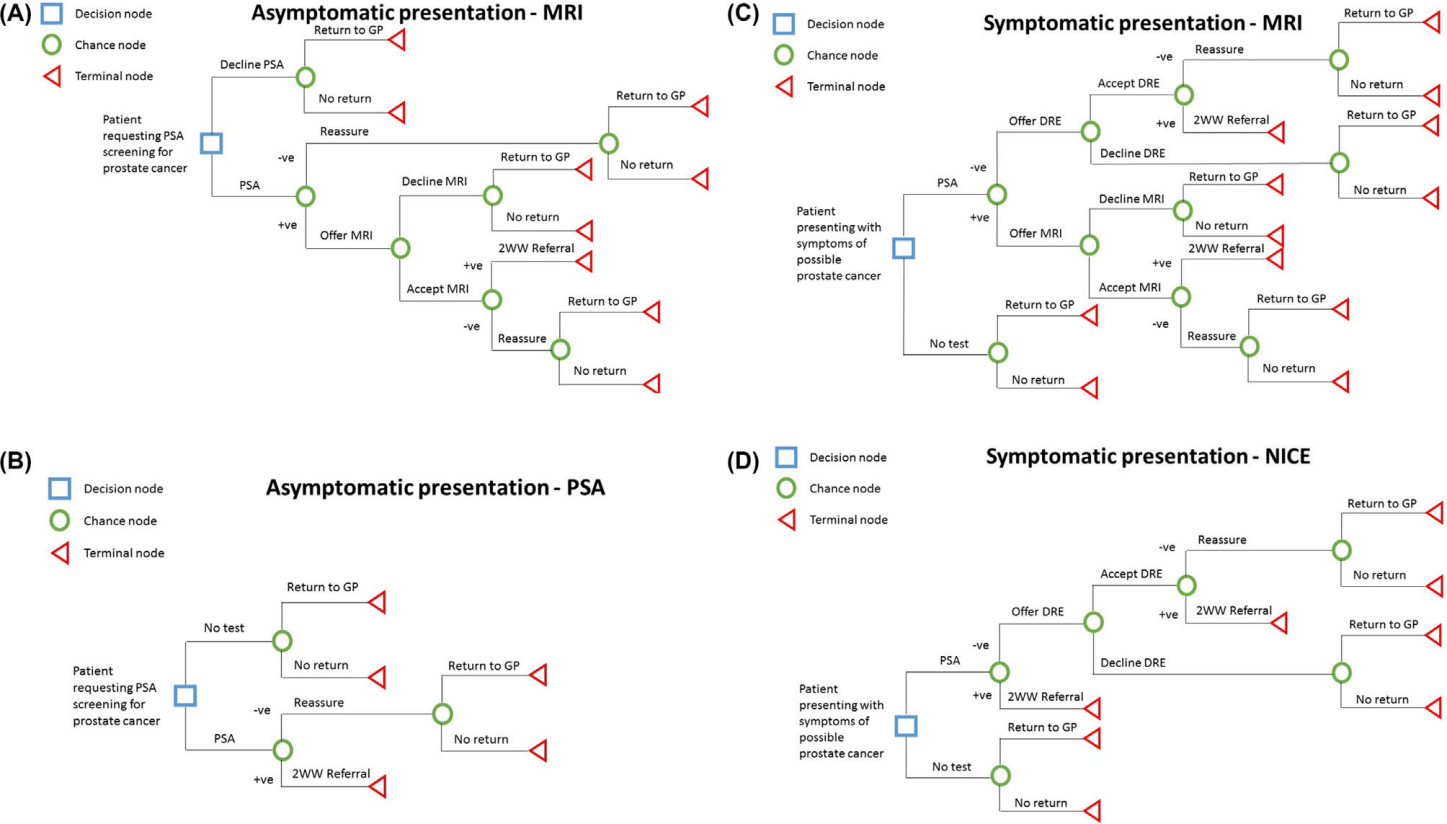
(A) Primary strategy integrating prostate MRI for patients presenting in primary care for opportunistic PSA screening. (B) Comparator strategy following usual care for patients presenting in primary care for opportunistic PSA screening. (C) Primary strategy integrating prostate MRI for symptomatic patients presenting in primary care. (D) Comparator strategy for symptomatic patients presenting in primary care following current NICE guidelines. PSA, prostate‐specific antigen; DRE, digital rectal exam’ MRI, magnetic resonance imaging; 2WW, 2‐week wait referral.

The main proposed patient benefits from integrating prostate MRI into the diagnostic pathway are increased detection of clinically significant prostate cancer, without diagnosing more cases of clinically insignificant prostate cancer, and safely avoiding biopsy procedures in patients with a low likelihood of clinically significant prostate cancer. A reduction in the number of prostate biopsies should reduce costs for the NHS, both from fewer biopsies being performed and a resulting reduction in complications such as urosepsis.^5^ The proposed pathway would require at least the same number of outpatient consultations before biopsy, or possibly more, and adds a further diagnostic test. Patients attending hospital outpatient appointments face several challenges, including transport, parking costs, and disruption to their usual activities.^6^ The NHS has significant capacity constraints with MRI scanner availability^7^ and diagnostic workforce shortages.^8^, ^9^ Furthermore, NHS urological cancer services were already struggling to meet NHS targets for time to diagnosis and time to commencing treatment prior to the publication of recommendations for implementing prostate MRI from NHS England and NICE.^10^

The current NHS prostate cancer diagnostic pathway employs prostate MRI as a secondary care test, as do other comparable healthcare systems. There are no known examples of primary care use of prostate MRI for the early detection of clinically significant prostate cancer. The only test for prostate cancer currently available to primary care clinicians is the prostate‐specific antigen (PSA) blood test. The increasing use of PSA is thought to have been a key contributing factor to the rise in the incidence of prostate cancer (more often lower‐risk disease) in high‐income countries in recent decades,^11^ and has not been clearly shown to reduce mortality when used as a screening test in asymptomatic patients.^12^ PSA has poor face validity with GPs owing to the perceived poor diagnostic accuracy; indeed, evidence on the diagnostic accuracy of PSA for clinically significant prostate cancer and in symptomatic patients is limited.^13^ Implementing a more accurate test for prostate cancer, such as prostate MRI, into primary care could have similar benefits to those found already in secondary care studies. It may have additional benefits for the NHS in terms of reducing waiting times in the prostate cancer diagnostic pathway, given that MRI has already been performed to inform the referral decision, therefore reducing urology referrals from primary care.

There are currently no published full economic evaluations of MRI‐based prostate cancer diagnostic pathways that consider the primary care elements of the pathway and no studies which have modelled the use of prostate MRI in a primary care setting.^14^ The aim of this early economic evaluation was to explore the potential impacts of incorporating prebiopsy MRI into primary care within the prostate cancer diagnostic pathway. The objectives were to explore the following questions:

1. What is the proportion of patients currently referred on the urgent suspected prostate cancer pathway following NICE guidance NG12 who are potentially referred without clinical benefit, and what proportion of prostate cancer cases are missed by the current primary care diagnostic pathway?
2. What are the expected differences in costs for the NHS and utility for patients between the existing primary care prostate cancer diagnostic pathway and a pathway employing prereferral prostate MRI in primary care?
3. What are the expected differences in costs and outcomes between using multiparametric MRI (mpMRI) and biparametric MRI (bpMRI) for prereferral MRI in the proposed primary care prostate cancer diagnostic pathway?
4. What are the expected differences in costs and outcomes in the proposed primary care prostate cancer diagnostic pathway between asymptomatic patients undergoing opportunistic screening for prostate cancer and symptomatic patients being investigated for prostate cancer?

## 2 SUBJECTS AND METHODS

The conceptualisation, construction and development of this decision analytic model have been undertaken following the process outlined by the ISPOR‐SMDM modelling good research practice task force.^15^

### 2.1 Conceptualising the model

There are two main routes through which most patients with prostate cancer will ultimately be diagnosed. The first relates to screening asymptomatic patients with a PSA test, then referring patients with a raised PSA above a recommended threshold. There are very few national, PSA‐based prostate cancer screening programmes globally due to the lack of evidence for a mortality benefit.^12^, ^16^ In the UK, patients can undergo opportunistic PSA screening for prostate cancer following an informed discussion with their GP about the potential benefits and harms involved.^17^ Estimates vary as to the level of PSA testing undertaken that is for opportunistic screening purposes in primary care.^18^, ^19^

The second main route through which patients are diagnosed with prostate cancer is following the development of lower urinary tract symptoms (LUTSs). The association between prostate cancer and LUTS is controversial,^20^ although most patients with prostate cancer report having symptoms such as LUTS prior to their diagnosis.^21^ NICE guideline NG12 ‘*Suspected cancer: recognition and referral*’ recommends GPs to consider a digital rectal examination (DRE) of the prostate and PSA test for any patients presenting with LUTS, visible haematuria or erectile dysfunction and to refer urgently for further investigation if either the DRE or PSA is abnormal.^22^

These two main routes to diagnosis were incorporated into a conceptual model of the prostate cancer diagnostic pathway (see Figure S1), which was developed with input from primary and secondary care clinicians, a patient and public involvement (PPI) group and a meeting of the CanTest International School in February 2021.^23^ The other much less common routes to diagnosis of prostate cancer include incidental abnormal findings on DRE for other purposes, discovery of a prostate cancer following a routine urology referral for noncancer reasons or late‐stage diagnosis through emergency presentation.^24^ These are not considered further in this study.

### 2.2 Modelling approach

A decision analytic modelling approach using decision trees was chosen for several reasons. Decision modelling allows the comparison of expected costs and outcomes for a range of options being considered for a particular problem, even when there is uncertainty around the decision(s).^25^, ^26^ Employing decision trees is a simple but effective method for decisions with shorter time horizons that captures the potential consequences of following different clinical pathways.^27^ Cost utility analysis was performed to compare the change in costs of implementing prostate MRI in primary care and patient utility from the diagnostic pathway and cancer diagnosis.

### 2.3 Disease

The primary disease of interest is prostate cancer. Clinically significant prostate cancer is defined based on the histology of the tumour using the Gleason scoring system (Gleason score ≥ 7 or Gleason grade group ≥ 2) and informs treatment and prognosis.^28^ Patients with localised clinically significant prostate cancer are generally offered invasive treatments, including radical prostatectomy or radiotherapy. Patients with clinically insignificant tumours (Gleason score = 6 or Gleason grade group = 1) are recommended to undergo active surveillance as there is a very low risk of tumour progression, cancer‐related morbidity or mortality.^4^ The diagnostic accuracy of mpMRI and bpMRI for clinically significant prostate cancer has been extensively researched.^29^, ^30^ However, the ability of DRE and PSA to discriminate between clinically significant and clinically insignificant prostate cancer is less well understood and assumed to be poor.^13^ Therefore, a diagnosis of any prostate cancer was used as an outcome for the models.

### 2.4 Perspective

A health system perspective was chosen for this model, that is, NHS and personal social services. Specifically, this model aimed to explore the potential impacts of adding primary care prostate MRI to the existing prostate cancer diagnostic pathway to inform the design of clinical pathways by NHS commissioners and cancer alliances. This approach is similar to that used by the NICE Health Technology Assessment reference case.^31^

### 2.5 Target population

The population of interest for this model includes male UK patients aged 50 years and over, either presenting to NHS primary care without urinary symptoms and requesting opportunistic PSA screening or presenting with symptoms of possible prostate cancer.

### 2.6 Strategies/comparators

The opportunistic prostate cancer screening pathway modelled starts with a patient aged 50 years and above who presents to their GP requesting an opportunistic PSA screening test. Current practice dictates that any patient with a raised PSA level is referred on the urgent suspected cancer (USC) pathway for further investigation (see Figure 2B). The primary strategy being assessed for this patient population involves a prostate MRI for any patient with an elevated PSA, and only entering the USC pathway if the MRI is reported as abnormal (see Figure 2A).

For a patient aged 50 years and above who presents to primary care for the first time with symptoms that may relate to an undiagnosed prostate cancer that are highlighted in NICE NG12 (LUTS, visible haematuria or erectile dysfunction), guidelines recommend that patients are offered a PSA test and DRE. In the current pathway (Figure 2D), patients with an abnormal result for either PSA or DRE receive a USC prostate cancer referral for outpatient prostate MRI and biopsy (if there is an abnormal finding on the MRI). The primary strategy assessed by this model would involve all patients with an abnormal PSA test result undergoing prostate MRI, and only entering the USC pathway if the MRI is abnormal (see Figure 2C). Patients with abnormal DRE would still be referred urgently without a subsequent MRI.^22^

### 2.7 Resources/costs

Resource costs used in the analyses of this model included staffing costs for clinicians in primary and secondary care involved with the pathways to diagnosis.^32^ Outpatient appointments, including two week wait consultations, were also considered.^33^ Tests used in primary care and diagnostic tests following referral were included as well.^34^

### 2.8 Time horizon

The time horizon considered for this model was 12 months following first presentation to primary care, a period chosen to reflect the focus on understanding the role of prostate MRI in the diagnosis of prostate cancer in primary care. A further reason for a relative short time horizon is the lack of evidence around the discriminative ability of symptoms, PSA and DRE for differentiating clinically significant from clinically insignificant prostate cancer, which is needed to confidently estimate likely treatments and long‐term outcomes following diagnosis.

### 2.9 Health outcomes

The primary outcome for this model is a diagnosis of prostate cancer. Diagnosis of clinically significant prostate cancers is important, but not used as an outcome, for reasons outlined above. Further relevant health outcomes captured by this model include the annual disutility experienced by patients from the various tests and stage at diagnosis, number of prostate MRI scans undertaken per year, proportion of patients referred for prostate biopsy unnecessarily due to false positive test results, and the proportion of patients with a missed diagnosis of prostate cancer.

### 2.10 Assumptions

Four assumptions have been made:

1. Test performance characteristics for PSA and MRI are similar in primary care to secondary care, as there are no primary care studies to estimate test accuracy in this clinical setting.
2. All patients in the cohort for the models have the same average prostate cancer risk.
3. All GPs refer patients in accordance with NICE guideline NG12 recommendations.
4. All referred patients would undergo the recommended transperineal prostate biopsy.

### 2.11 Linked data approach

This model employed a linked data approach from a range of data sources, including observational studies, diagnostic test accuracy studies, cost‐effectiveness analyses, systematic reviews and analyses of existing datasets. This approach was necessary as there is no existing primary care trial of prostate MRI upon which to base an economic evaluation.

### 2.12 Sensitivity analyses

Analyses were performed within the MRI models using mpMRI and bpMRI separately to assess for noninferiority of bpMRI. One‐way deterministic sensitivity analysis (DSA) estimated the effect of the uncertainty around baseline estimates for the parameters included in the models, using the 95% confidence intervals in the included studies and additional analyses (see Table 1). A tornado diagram summarised the DSA results showing the impact of uncertainty for individual model parameters. Probabilistic sensitivity analysis (PSA) was also performed, using beta distributions for probabilities and utilities and gamma distributions for costs. PSA was run for 1000 cycles of the model, and results presented using cost‐effectiveness scatter plots and cost‐effectiveness acceptability curves.

**TABLE 1:** Probabilities, costs, and utilities used in the model.

Parameter	Baseline estimate (95% CI)	Source
Undergoing DRE	0.62 (0.57, 0.66)	Young et al.^35^
DRE sensitivity	0.29 (0.25, 0.32)	Jones et al.^36^
DRE specificity	0.91 (0.89, 0.92)	Jones et al.^36^
Undergoing PSA for symptoms	0.84 (0.80, 0.87)	Young et al.^35^
Undergoing PSA screening	0.017 (0.016, 0.017)	Clift et al.^18^
PSA sensitivity (symptomatic)	0.93 (0.88, 0.96)	Merriel et al.^37^
PSA specificity (symptomatic)	0.20 (0.12, 0.33)	Merriel et al.^37^
PSA sensitivity (screening)	0.69 (0.58, 0.78)	Ilic et al.^12^
PSA specificity (screening)	0.56 (0.50, 0.62)	Ilic et al.^12^
Undergoing MRI	0.98 (0.96, 0.99)	Ahmed et al.^1^
mpMRI sensitivity	0.91 (0.83, 0.95)	Drost et al.^30^
mpMRI specificity	0.37 (0.29, 0.46)	Drost et al.^30^
bpMRI sensitivity	0.87 (0.78, 0.93)	Bass et al.^29^
bpMRI specificity	0.72 (0.56, 0.84)	Bass et al.^29^
Returning with symptoms	0.14 (0.13, 0.14)	Supplementary file 3
Returning for repeat screening	0.20 (0.19, 0.20)	Young et al.^19^
**Cost**	**Amount (£)**	**Source**
GP appointment	£33.00	Curtis and Burns^32^
Nurse appointment	£8.17	Curtis and Burns^32^
PSA	£5.91	Ramsay et al.^34^
mpMRI (direct access) RD03z	£190.00	NHS ref costs^33^
mpMRI (outpatient) RD03Z	£217.00	NHS ref costs^33^
bpMRI (direct access) RD01A	£121.00	NHS ref costs^33^
bpMRI (outpatient)RD01A	£143.00	NHS ref costs^33^
USC appointment WF01B	£144.00	NHS ref costs^33^
TRUS biopsy LB67Z	£504.00	NHS ref costs^33^
Transperineal template biopsy LB77Z	£1413.00	NHS ref costs^33^
**Health state**	**Annual disutility (range)**	**Source**
DRE	0.00019 (0, 0.00019)	Assumption
PSA	0.00019 (0, 0.00019)	Barnett et al.^38^
MRI	0.00077 (0.00038, 0.00012)	Barnett et al.^38^
Biopsy	0.00577 (0.00346, 0.0075)	Barnett et al.^38^
Post biopsy infection	0.0161 (0.00969, 0.0291)	Barnett et al.^38^
Early‐stage diagnosis	0.0167 (0.0125, 0.0208)	Barnett et al.^38^
Delayed diagnosis	0.3 (0.3, 0.38)	Barnett et al.^38^
Late‐stage diagnosis	0.4 (0.14, 0.76)	Barnett et al.^38^

Abbreviations: DRE, digital rectal examination; PSA, prostate‐specific antigen; MRI, magnetic resonance imaging; mpMRI, multiparametric MRI; bpMRI, biparametric MRI; USC, urgent suspected cancer referral; TRUS, transrectal ultrasound guided; CI, confidence interval.

### 2.13 Evidence sources

An outline of the evidence sources used for this study and quality appraisal can be found in the Supplementary file 2. The following baseline estimates were generated for use in the modelling undertaken in this study (see Table 1).

### 2.14 Analysis software

The analyses were conducted using Stata version 17.0 (StataCorp. 2021. Stata Statistical Software: Release 17, College Station, TX, StataCorp LLC) and Microsoft Excel (Microsoft Corporation 2021).

## 3 RESULTS

### 3.1 Model outputs

#### 3.1.1 Base case analysis

**FIGURE 3:**
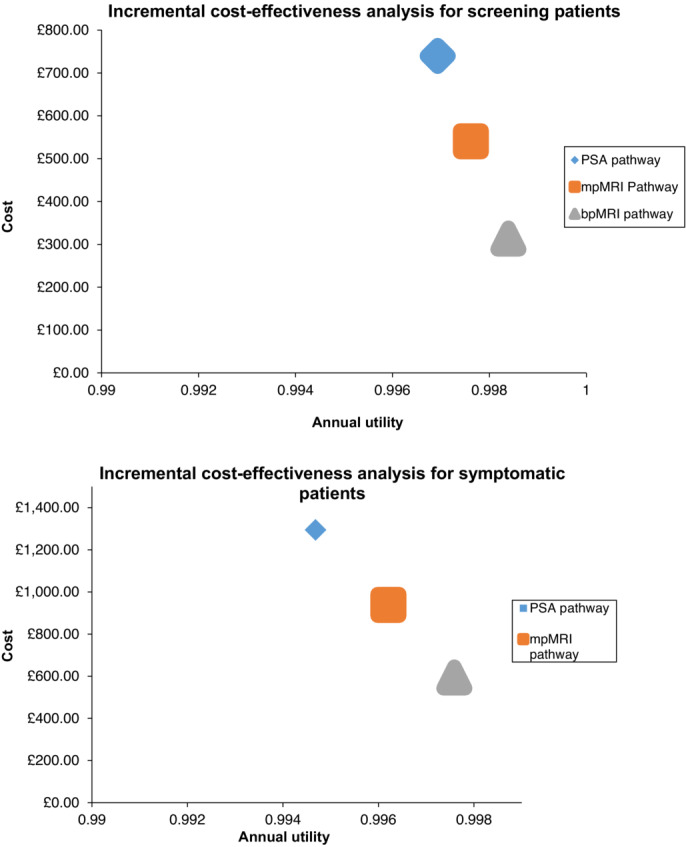
Incremental cost‐effectiveness of PSA, mpMRI, and bpMRI pathways for screening and symptomatic patients. PSA, prostate‐specific antigen; mpMRI, multiparametric MRI; bpMRI, biparametric MRI.

Table 2 shows the incremental costs and utilities of the mpMRI and bpMRI pathways compared with the PSA pathway. This was dominated by both MRI‐based pathways for both symptomatic patients and patients undergoing opportunistic screening. bpMRI pathways had more significant efficiencies in cost and utility than mpMRI pathways in both patient groups. Figures 2 and 3 demonstrate these results graphically.

**TABLE 2:** Costs and utilities of each strategy assessed in the base case analysis. Incremental costs and utilities for the mpMRI and bpMRI pathways were compared with the PSA pathway and were dominant compared with the PSA pathway for symptomatic and screening patients.

Strategy	Costs	Annual utility	Incremental costs (relative to PSA)	Incremental utility
Base case—symptomatic patients				
PSA pathway	£1294.22	0.9946824		
mpMRI pathway	£938.42	0.9962101	− £355.80	0.0015277
bpMRI pathway	£594.46	0.9975885	− 699.77	0.0029060
Base case—screening patients				
PSA pathway	£739.90	0.9969314		
mpMRI pathway	£540.56	0.9976161	− £199.33	0.0006847
bpMRI pathway	£313.60	0.9983909	− £426.30	0.0014595

Abbreviations: PSA, prostate‐specific antigen; mpMRI, multiparametric MRI; bpMRI, biparametric MRI.

Estimates of the potential impact of implementing primary care prostate MRI showed that significantly more MRI scans would be needed compared with the current pathway for both symptomatic (66 626 scans per annum vs. 37 456 scans per annum) and screening (36 139 scans per annum vs. 20 324 scans per annum) pathways per 100 000 patients. USC referrals would reduce for both mpMRI (38% for symptomatic and screening patients) and bpMRI (71% for symptomatic and 72% for screening) pathways. More patients would experience a missed diagnosis of prostate cancer in MRI‐based pathways than the PSA pathway (Table 3) owing to false negatives from MRI.

**TABLE 3:** Predicted effects of different prostate cancer diagnostic pathway strategies per 100 000 men.

Strategy	Number of MRI scans done	Number of USC referrals	Number of missed diagnoses per 100 000 patients
Base case—symptomatic patients			
PSA pathway	37 456	68 103	36
mpMRI pathway	66 626	42 367	50
bpMRI pathway	66 626	19 426	56
Base case—screening patients			
PSA pathway	20 324	36 952	50
mpMRI pathway	36 139	22 795	83
bpMRI pathway	36 139	10 176	87

Abbreviations: PSA, prostate‐specific antigen; MRI, magnetic resonance imaging; mpMRI, multiparametric MRI; bpMRI, biparametric MRI; USC, urgent suspected cancer.

### 3.2 Sensitivity analyses

Deterministic sensitivity analyses varying each parameter by the upper and lower limits of the 95% CIs showed no significant change in the dominance of the MRI‐based prostate cancer diagnostic pathways in the models for patients for opportunistic screening or symptomatic patients (tornado plots in Supplementary file 4).

Probabilistic sensitivity analyses showed no significant change in estimated and incremental costs for the MRI pathways but suggested small incremental utility deficits relative to the PSA pathway (Supplementary file 5). Cost‐effectiveness planes show the bpMRI pathway is more often below the current NICE threshold of £30 000 compared with mpMRI for both screening and symptomatic patients (Supplementary file 6).

Cost‐effectiveness acceptability curves suggested that MRI pathways were more likely to be cost‐effective than PSA pathways regardless of the willingness‐to‐pay threshold, with a higher probability for bpMRI than mpMRI (Supplementary file 7).

## 4 DISCUSSION

### 4.1 Main findings

This early economic evaluation of integrating prostate MRI into primary care within the prostate cancer diagnostic pathway strongly suggests that it is more cost‐effective than the current practice of relying on PSA alone as a triage test, both in opportunistic screening and symptomatic patients. Using prostate MRI in primary care to select urgent referrals for prostate cancer biopsy would result in a greater reduction in referrals, relative to additional prostate MRIs, with a small increase in the number of men with a missed diagnosis. bpMRI was more likely to be cost effective than mpMRI when each was compared with the current standard of care. Sensitivity analyses were consistent with the base case analysis for costs of the different pathways compared but found a small utility decrement with MRI pathways (as opposed to a small utility gain in the base case analysis).

### 4.2 Comparison to existing literature

No known study has modelled the estimated costs and utilities of the current primary care prostate cancer diagnostic pathway, nor considered the potential impact of prostate MRI on the pathway. All existing cost‐effectiveness reports of prebiopsy MRI‐based prostate cancer diagnostic pathways use secondary care populations. Some published cost‐effectiveness analyses focus on the use of prostate MRI within prostate cancer screening, using PSA to identify men at higher risk of prostate cancer,^38^, ^39^, ^40^ whilst others consider the effect of prostate MRI for patients referred on the basis of abnormal DRE and/or PSA.^41^, ^42^, ^43^, ^44^ Consistent with the results of this study, in both scenarios, the use of prebiopsy MRI was found to be more cost‐effective than the current pathway. The specific biopsy approach was kept constant in this study, in contrast to other published economic evaluations where different biopsy approaches have been compared as part of the MRI‐based diagnostic strategies.

### 4.3 Strengths and weaknesses

This early economic evaluation has several strengths. A simple decision model was employed over a fixed time horizon to generate early estimates for the potential impact of an as‐yet untested test in a primary care setting. Such an approach is appropriate to improve the interpretation of the findings and the reproducibility of the research. A linked data approach was undertaken to inform the model. This was necessary in the absence of a relevant trial of prostate MRI in primary care, but also allowed the integration of multiple data sources to generate more robust analyses. Sensitivity analyses broadly supported the base case findings, strengthening the confidence in the model outputs.

However, there are several limitations. Prostate MRI is not currently used in primary care in any country, and there is no research evidence in this healthcare setting, requiring assumptions to be made about extrapolating the performance of the test to a primary care setting. Most of the other data used to inform study parameters was also generated in secondary care. Most relevant studies for PSA for prostate cancer detection in patients with symptoms have a high risk of bias and probably overestimate the accuracy of PSA.^37^ There is also no evidence for the accuracy of PSA, nor for other clinical features used in primary care to identify patients with suspected prostate cancer such as a DRE, in detecting clinically significant prostate cancer. This makes estimating the impacts of changes to the primary care prostate cancer diagnostic on treatments and long‐term outcomes difficult.

### 4.4 Implications for practice

Evidence from clinical trials in the UK^1^, ^2^, ^45^, ^46^ and other high‐income countries show that prebiopsy prostate mpMRI is accurate in the detection of clinically significant prostate cancer. Prostate MRI also provides valuable information for guiding prostate biopsy. There is growing evidence that bpMRI is noninferior to mpMRI in the detection of clinically significant prostate cancer and is a quicker and cheaper test without requiring intravenous contrast.^29^ Prostate MRI is currently only used in secondary and tertiary care settings. By comparison, the limited evidence base for tests for prostate cancer detection such as PSA and DRE suggest that they perform less well when used in primary care, and it is not known whether these tests are able to accurately distinguish between clinically significant and clinically insignificant prostate cancer, with important implications for prostate cancer detection, treatment decisions, and patient outcomes.

This modelling study suggests that integrating prostate MRI into the primary care diagnostic pathway could have benefits for patients and health services in the form of fewer urgent suspected cancer referrals and subsequent prostate biopsies, with a small increase in the number of missed prostate cancer diagnoses. It is unknown what proportion of the missed diagnoses would be clinically significant prostate cancer, although current evidence suggests that the risk of false negative MRI for clinically significant disease is low.^47^ There would be a significant increase in the number of MRI scans ordered, which is potentially a challenge for the NHS to implement with existing MRI scanner capacity shortfalls^7^ and an insufficient diagnostics workforce.^8^, ^9^

Direct access for primary care clinicians to cancer diagnostic tests is established for other cancer types, including upper and lower gastrointestinal cancers,^48^ and may be appropriate for prostate cancer. Primary evidence for the accuracy of prostate MRI ordered from and actioned in primary care, and better evidence for the accuracy and effectiveness of existing tests as they are currently used in the NHS, is clearly needed.

## AUTHOR CONTRIBUTIONS

S. W. D. M., A. E. S., F. M. W., and W. H. conceived the study and its aims. S. W. D. M. performed the evidence gathering and synthesis. S. W. D. M. and S. J. P. extracted, prepared, and analysed the CPRD data for repeat consultations (Supplementary File 3). S. W. D. M. and N. B. C. extracted, prepared, and analysed the South‐West Peninsula Cancer Alliance Prostate Cancer Dashboard data. S. W. D. M. and A. E. S. undertook the model development and decision analytic modelling. P. B. was a member of the PPI group that supported the study. S. W. D. M. prepared the first draft of the manuscript. All authors reviewed and inputted into the draft manuscript and approved the final version for publication.

## CONFLICT OF INTEREST STATEMENT

The authors have no declarations of interest to make in relation to this study.

## FUNDING INFORMATION

This study was supported by the CanTest Collaborative (funded by Cancer Research UK C8640/A23385) of which F.M.W. and W.H. were co‐Directors, A. E. S. was a co‐investigator, and S. W. D. M. was a researcher. The CPRD dataset used to calculate some of the model parameters was supported by the IMPACT study (CRUK C56843/A21550). SWDM is supported by the NIHR Manchester Biomedical Research Centre (NIHR203308). The funder of the study had no role in study design, data collection, data analysis, data interpretation, or writing of the report. The views expressed are those of the author(s) and not necessarily of the NIHR or the Department of Health and Social Care.

## ETHICS STATEMENT

No primary data collection was undertaken for this study. All data sources were either publicly available or already had ethical approval in place.

## Supporting information


**Figure S1.** Conceptual model of the existing prostate cancer diagnostic pathway
**Table S2.**1. Quality appraisal of systematic reviews used for parameter estimates using AMSTAR‐2 (Y – Yes; N – No; NA – Not Applicable; H – High; M – Moderate; L – Low; CL – Critically Low)(41)
**Table S2.**2. Study quality assessment of observational studies using MINORS (2 – reported and adequate; 1 – reported, not adequate; 0 – not reported; Red – low quality; yellow – medium quality; green – high quality)(42)

**Table S2.3.** Quality assessment of model‐based economic evaluations using the Philips framework(44) (Green – low risk of bias; Yellow – some risk of bias; Red – high risk of bias)

**Table S3.1.** Included participant demographics
**Table S3.**2. Variable descriptions
**Table S3.**3. Proportions of index presentation types for patients with prostate cancer
**Table S3.**4. Initial PSA results for patients with prostate cancer
**Table S3.**5. Diagnostic interval (DI) for patients by number of pre‐referral consultations
**Table S3.**6. Proportion of patients with 3 + pre‐referral consultations overall, and within time periods relating to NICE suspected cancer guidelines.

**Figures S4.1 & S4.2.** Tornado plots for deterministic sensitivity analyses in screening patients
Figures S4.3 & S4.4. Tornado plots for deterministic sensitivity analyses in symptomatic patients

**Table S5.** Probabilistic sensitivity analyses of estimated costs and utilities associated with the three strategies in the model

**Figure S6.1 & S6.2.** Cost‐effectiveness scatter plots for screening patients
**Figure S6.**3 & S6.4. Cost‐effectiveness scatter plots for symptomatic patients

**Figure S7.1 & S7.2.** Cost‐effectiveness acceptability curves for screening patients
**Figure S7.**3 & S7.4. Cost‐effectiveness acceptability curves for symptomatic patients

## Data Availability

Data used for this model from published papers are freely available to access. CPRD data from the CRUK IMPACT study and South‐West Peninsula Cancer Alliance Prostate Cancer Dashboard data are not publicly available; any requests for access should be made to S. J. P. and N. B. C., respectively.
