# Supplementary material for: Early economic evaluation of magnetic resonance imaging for prostate cancer detection in primary care
Source: BJUI Compass. 2024 Jul 10;5(9):855–64. doi: 10.1002/bco2.409 (PMC11420105; doi:10.1002/bco2.409)
Supplement: Supplementary file 1 — Figure S1. Conceptual model of the existing prostate cancer diagnostic pathway Table S2.1. Quality appraisal of systematic reviews used for parameter estimates using AMSTAR‐2 (Y – Yes; N – No; NA – Not Applicable; H – High; M – Moderate; L – Low; CL – Critically Low)(41) Table S2.2. Study quality assessment of observational studies using MINORS (2 – reported and adequate; 1 – reported, not adequate; 0 – not reported; Red – low quality; yellow – medium quality; green – high quality)(42) [file BCO2-5-855-s006.docx]

Supplementary file 1


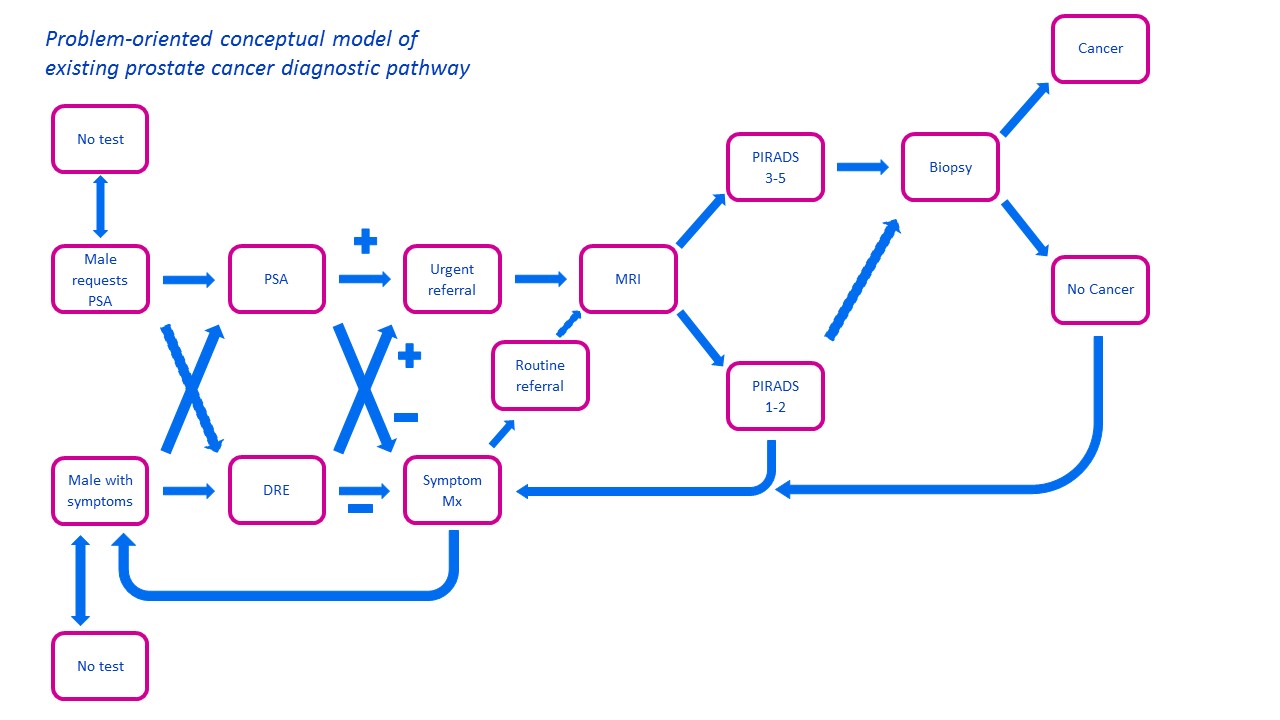


Figure S1 – Conceptual model of the existing prostate cancer diagnostic pathway

**+** indicates an abnormal/positive result; **–** indicates a normal/negative result

PSA – Prostate Specific Antigen; DRE – Digital Rectal Examination; Mx – management; MRI – Magnetic Resonance Imaging; PIRADS – Prostate Imaging-Report and Data System
