# Supplementary material for: Early economic evaluation of magnetic resonance imaging for prostate cancer detection in primary care
Source: BJUI Compass. 2024 Jul 10;5(9):855–64. doi: 10.1002/bco2.409 (PMC11420105; doi:10.1002/bco2.409)
Supplement: Supplementary file 2 — Table S2.3. Quality assessment of model‐based economic evaluations using the Philips framework(44) (Green – low risk of bias; Yellow – some risk of bias; Red – high risk of bias) [file BCO2-5-855-s007.docx]

Supplementary file 2

**Bass EJ, Pantovic A, Connor M, *et al*. A systematic review and meta-analysis of the diagnostic accuracy of biparametric prostate MRI for prostate cancer in men at risk. Prostate Cancer Prostatic Dis. Published online 2020. doi:10.1038/s41391-020-00298-w**

This systematic review and meta-analysis updated a recent review examining the diagnostic performance of biparametric magnetic resonance imaging (bpMRI) for the detection of any prostate cancer and clinically significant prostate cancer, and compared the performance of bpMRI to multiparametric MRI (mpMRI). This review identified 11 new studies since the previous review on this subject was published and found a pooled sensitivity of 0.87 (95% CI 0.78, 0.93) and pooled specificity of 0.72 (95% CI 0.56, 0.84) for clinically significant prostate cancer. The AUC was 0.87. Meta-regression found no significant difference between the performance of bpMRI and mpMRI. The majority of studies were assessed as having a low risk of bias, although 16/45 studies had a high risk of bias for the ‘patient selection’ domain of QUADAS-2.

**Drost FJ, Osses DF, Nieboer D, Bangma CH, Steyerberg EW, Roobol MJ, *et al*. ‘Prostate MRI, with or without targeted biopsy and standard biopsy for detecting prostate cancer: A Cochrane systematic review and meta-analysis’. *Cochrane Database Syst Rev*. 2019;(4):CD012663.**

This Cochrane systematic review and meta-analysis aimed to determine the diagnostic accuracy of prostate MRI, MRI-targeted biopsy, an MRI pathway (prostate MRI with MRI-targeted biopsy for patients with a positive MRI), and systematic TRUS biopsy compared to template-guided biopsy (reference standard) for the detection of clinically significant prostate cancer. Pooled sensitivity of MRI was found to be higher than TRUS biopsy (0.91 95% CI 0.83, 0.95 vs 0.63 95% CI 0.19, 0.93) but with a lower specificity (0.37 95% CI 0.29, 0.46 vs 1.00 95% CI 0.91, 1.00). The MRI pathway had the most favourable diagnostic accuracy (pooled sens 0.72 95% CI 0.60, 0.82, pooled spec 0.96 95% CI 0.94, 0.98) of all methods assessed. The authors rated the quality of evidence as low and recommended further development of new prostate cancer diagnostic pathways incorporating MRI.

**Ilic D, Djulbegovic M, Jung JH, Hwang EC, Zhou Q, Cleves A, *et al*. ‘Prostate cancer screening with prostate-specific antigen (PSA) test: a systematic review and meta-analysis’. *BMJ*. 2018;362:k3519.**

This systematic review and meta-analysis was written as an update of a Cochrane review following the publication of the findings of the Cluster Randomized Trial of PSA testing for Prostate cancer (CAP)(37). The review sought to assess the latest evidence on the potential benefits and harms of PSA screening for prostate cancer. The primary outcomes related to all-cause and prostate cancer specific mortality, for which there was no clear evidence of an effect of PSA screening. The study also reported estimates for the false positive screening rate (67%, high quality evidence) and false negative screening rate (15%, low quality evidence), from which estimates were generated for the model(8). A 2x2 table was imputed from estimated false-positive and false-negative rates reported in the paper, and exact confidence intervals calculated using the Clopper-Pearson method.

**Jones D, Friend C, Dreher A, Allgar V, Macleod U. ‘The diagnostic test accuracy of rectal examination for prostate cancer diagnosis in symptomatic patients: a systematic review’. *BMC Fam Pract.* 2018;19:79.**

This systematic review and meta-analysis aimed to assess the evidence for the diagnostic accuracy of digital rectal examination (DRE) for the detection of prostate cancer in symptomatic patients presenting to primary care. Four studies with 3,225 patients were included; all were assessed to be of high methodological quality with significant heterogeneity. The pooled sensitivity of DRE for prostate cancer was found to be 0.29 (95% CI 0.25, 0.32) and the pooled specificity was 0.91 (95% CI 0.89, 0.92).

**Young SM, Bansal P, Vella ET, Finelli A, Levitt C, Loblaw A. ‘Systematic review of clinical features of suspected prostate cancer in primary care’. *Can Fam Physician*. 2015;61(1):e26–35.**

This systematic review and narrative synthesis sought to provide an update to evidence included in national guidelines for prostate cancer diagnosis in primary care. The authors identified two UK studies reporting the proportion of referred hospital patients with suspected prostate cancer that had a recorded DRE performed in primary care varied from 32% (48/148) to 77% (221/287), and the proportion with a pre-referral PSA result varied from 74% (211/287) to 97% (144/148). These studies were small, single-centre, retrospective observational studies. These samples were combined to generate a single proportion estimate with SE and 95% confidence intervals.

**Ahmed HU, Bosaily AE-S, Brown LC, Gabe R, Kaplan R, Parmar MK, *et al*. ‘Diagnostic accuracy of multi-parametric MRI and TRUS biopsy in prostate cancer (PROMIS): a paired validating confirmatory study’. *Lancet*. 2017 Jan 19;380:1–8.**

The PROMIS trial was a prospective, multi-centre, paired-cohort, confirmatory study comparing the diagnostic accuracy of multiparametric magnetic resonance imaging (mpMRI) to Transrectal Ultrasound guided (TRUS) biopsy, using template prostate mapping (TPM) biopsy as the reference standard. The PROMIS trial was included in the Cochrane review by Drost *et al*(28), and the sensitivity (0.93 95% CI 0.88, 0.96) and specificity (0.41 95% CI 0.36, 0.46) in the trial were not dissimilar to the pooled findings of the review. 97.8% (723/740) of potentially eligible participants underwent mpMRI in the PROMIS trial.

**Clift AK, Coupland C, Hippisley-Cox J. ‘Prostate-specific antigen testing and opportunistic prostate cancer screening: a cohort study in England’. *Br J Gen Pract*. 2021;71(703):e157–65.**

This cohort study of 3,211,276 patients from 1,457 GP practices within the QResearch database aimed to estimate the cumulative incidence of PSA testing and opportunistic PSA screening in UK GP practices between 1998 and 2017. Included patients had to have no previous PSA testing or history of prostatic disease, aged 40 years and above, and registered with an included GP practice for at least 12 months. The cumulative risk for a patient having at least one PSA test within 12 months of follow-up for any reason was 2.28% (95% CI 2.23, 2.32); and the cumulative risk of opportunistic PSA screening in the same time period was 1.67% (95% CI 1.66, 1.69).

**Young GJ, Harrison S, Turner EL, Walsh EI, Oliver SE, Ben-Shlomo Y, *et al*. ‘Prostate-specific antigen (PSA) testing of men in UK general practice: A 10-year longitudinal cohort study’. *BMJ Open*. 2017;7(10).**

This retrospective cohort study of 450,000 patients from within the Clinical Practice Research Datalink (CPRD) database was similar to Clift *et al*, in that the authors aimed to estimate the cumulative risk of PSA testing in UK primary care patients without a diagnosis of prostate cancer over a 10-year period. Unlike Clift *et al*(16), the study by Young *et al* assessed the risk of repeat testing and factors associated with repeat testing. They found that 20% (17,775 / 90,252) of patients with at least 12-months follow-up attended for a repeat test, which was more likely to occur for patients with a higher initial PSA test result.

**Barnett CL, Davenport MS, Montgomery JS, Wei JT, Montie JE, Denton BT. ‘Cost-effectiveness of magnetic resonance imaging and targeted fusion biopsy for early detection of prostate cancer’. *BJU Int*. 2018 Jul;122(1):50–8.**

This cost-effectiveness analysis study employed a validated, partially observable Markov model to estimate outcomes for PSA screening programmes that include pre-biopsy prostate MRI. The model employed published estimates for annual disutility relating to diagnostic tests and health states within the five screening strategies that were compared. This study found using pre-biopsy prostate MRI was cost-effective assuming a willingness-to-pay threshold of $100,000.

Risk of bias for the selected systematic review was assessed using the AMSTAR-2 critical appraisal tool(41). Four out of five reviews were graded as low confidence due to not reporting on the funding sources of included studies in the respective reviews. The review by Young *et al* was assessed as critically low confidence as the review team did not utilise two reviewers to independently identify included studies, in addition to not reporting individual study funding sources (see table 5.1). Please see the tables below for quality assessment of the studies described above. The three selected observational studies were assessed as high quality using the MINORS checklist(42) (see table 5.2), and the cost-effectiveness analysis by Burnett *et al* was found to have a low risk of bias on a majority of domains in the Philips framework(43) (see table 5.3).

| **Author** | PCIO question | Protocol followed | Design selected | Search strategy | Two reviewers | Two data extractors | Exclusions justified | Describe studies | Risk of bias | Funding sources | Meta-analysis | RoB impact | RoB interpreted | Heterogeneity discussed | Publication bias | Conflict of interest | Overall confidence |
| --- | --- | --- | --- | --- | --- | --- | --- | --- | --- | --- | --- | --- | --- | --- | --- | --- | --- |
| Bass  2020 | Y | Y | N | Y | Y | Y | Y | Y | Y | N | Y | Y | Y | Y | N | Y | L |
| Drost  2019 | Y | Y | Y | Y | Y | Y | Y | Y | Y | N | Y | Y | Y | Y | N | N | L |
| Ilic  2018 | Y | Y | Y | Y | Y | Y | Y | Y | Y | Y | Y | Y | Y | Y | N | Y | L |
| Jones  2018 | Y | Y | N | Y | Y | Y | Y | Y | Y | N | Y | N | Y | Y | N | Y | L |
| Young  2015 | N | N | Y | Y | N | N | Y | Y | Y | N | NA | NA | NA | Y | N | Y | CL |

Table S2.1 – Quality appraisal of systematic reviews used for parameter estimates using AMSTAR-2 (Y – Yes; N – No; NA – Not Applicable; H – High; M – Moderate; L – Low; CL – Critically Low)(41)

| **Author** | Aim | Consecutive pts | Prospective data | Endpoints | Unbiased assess | Fup appropriate | Loss to fup | Size calc | **Subtotal** | Adequate control | Contemporary | Equal groups | Analysis | **Total** |
| --- | --- | --- | --- | --- | --- | --- | --- | --- | --- | --- | --- | --- | --- | --- |
| Ahmed  2017 | 2 | 2 | 2 | 2 | 2 | 0 | 2 | 2 | 14 |  |  |  |  |  |
| Clift  2021 | 2 | 2 | 1 | 2 | 1 | 2 | 2 | 2 | 14 |  |  |  |  |  |
| Young  2017 | 2 | 2 | 1 | 2 | 1 | 2 | 2 | 2 | 14 |  |  |  |  |  |

Table S2.2 **–** Study quality assessment of observational studies using MINORS (2 – reported and adequate; 1 – reported, not adequate; 0 – not reported; Red – low quality; yellow – medium quality; green – high quality)(42)

| *Domains* | *Structure* | | | | | | | | | *Data* | | | | | | | | | | | *Consistency* | |
| --- | --- | --- | --- | --- | --- | --- | --- | --- | --- | --- | --- | --- | --- | --- | --- | --- | --- | --- | --- | --- | --- | --- |
| **Author** | **S1** | **S2** | **S3** | **S4** | **S5** | **S6** | **S7** | **S8** | **S9** | **D1** | **D2** | **D2a** | **D2b** | **D2c** | **D3** | **D4** | **D4a** | **D4b** | **D4c** | **D4d** | **C1** | **C2** |
| Burnett 2018 |  |  |  |  |  |  |  |  |  |  |  |  |  |  |  |  |  |  |  |  |  |  |

Table S2.3 – Quality assessment of model-based economic evaluations using the Philips framework(44) (Green – low risk of bias; Yellow – some risk of bias; Red – high risk of bias)
