# Supplementary material for: Early economic evaluation of magnetic resonance imaging for prostate cancer detection in primary care
Source: BJUI Compass. 2024 Jul 10;5(9):855–64. doi: 10.1002/bco2.409 (PMC11420105; doi:10.1002/bco2.409)
Supplement: Supplementary file 3 — Table S3.1. Included participant demographics Table S3.2. Variable descriptions Table S3.3. Proportions of index presentation types for patients with prostate cancer Table S3.4. Initial PSA results for patients with prostate cancer Table S3.5. Diagnostic interval (DI) for patients by number of pre‐referral consultations Table S3.6. Proportion of patients with 3 + pre‐referral consultations overall, and within time periods relating to NICE suspected cancer guidelines. [file BCO2-5-855-s003.docx]

Supplementary file 3

Secondary analysis of the CRUK IMPACT study Clinical Practice Research Datalink (CPRD) dataset

**Aim**

To estimate the probability of a patient presenting in primary care with lower urinary tract symptoms and an undiagnosed prostate cancer returning for repeat consultations when not referred for investigation at initial presentation.

**Methods**

*Data source*

This analysis utilised a Clinical Practice Research Datalink (CPRD) dataset that has been used for the Cancer Research UK-funded IMPACT study. The CRUK IMPACT study aimed to estimate diagnostic intervals and patient outcomes for 22 cancer sites, including prostate cancer, and assess the impact of revisions made to National Institute for Health and Care Excellence (NICE) guidelines for GPs on suspected cancer referral. CPRD is a large, anonymised, primary care dataset consisting of coded data from primary healthcare records extracted by GP practices across the UK, including over 16 million currently registered patients. CPRD has been shown to be representative of the UK population.

*Population*

41,115 patients in CPRD with a diagnosis of prostate cancer between 01/01/2000 and 31/12/2017 were included in this dataset. The mean age at diagnosis for these patients was 72.11 years (+/- 9.42 years). See table S4.1 below for further information about geographical region and deprivation for the practice area from which the patients come from. Complete consultation data was available for 34,409 patients in this cohort (83.7%).

| Socioeconomic status | |
| --- | --- |
| **Townsend deprivation index** | **N (%)** |
| 1 | 7,111 (17.28%) |
| 2 | 6,633 (16.12%) |
| 3 | 5,083 (12.35%) |
| 4 | 3,959 (9.62%) |
| 5 | 1,985 (4.82%) |
| Missing | 16,384 (39.81%) |
| Geography | |
| **Region** | **N (%)** |
| North East | 549 (1.33%) |
| North West | 4,217 (10.25%) |
| Yorkshire & Humber | 1,141 (2.77%) |
| East Midlands | 1,081 (2.63%) |
| West Midlands | 4,000 (9.72%) |
| East of England | 3,428 (8.33%) |
| South West | 3,959 (9.62%) |
| South Central | 4,643 (11.28%) |
| London | 3,350 (8.14%) |
| South East Coast | 4,835 (11.75%) |
| Northern Ireland | 1,311 (3.19%) |
| Wales | 3,406 (8.28%) |
| Scotland | 5,235 (12.72%) |

Table S3.1 – Included participant demographics

Consultation data, including date of consultation and coded reason for contact with primary care, and PSA testing data, including the date of test and result, was available from 01/01/1999 to 31/12/2017.

*Data access*

This CPRD dataset received regulatory and ethical approval from the Medicines and Healthcare products Regulatory Agency (MHRA) Independent Scientific Advisory Committee (ISAC) (ISAC protocol number 16_037A2). The analysis performed was within the remit of the ISAC approval given, and therefore no amendment to the existing approval was necessary.

*Data preparation*

Analysis datasets from the CRUK IMPACT study covering baseline patient data, consultation data, and PSA testing data were combined using the CPRD *epatid* variable for the purposes of this analysis. Duplicate entries were removed. See Table S4.2 for a list of key variables that were prepared for the analysis.

| **Variable name** | **Description** |
| --- | --- |
| epatid | Individual patient ID from CPRD |
| age_dx | Patient age at diagnosis (years) |
| region | Geographical region of patient’s GP practice |
| townsend2001_5 | Townsend Index of Deprivation for patient’s GP practice |
| prostate_diagdate | Date of diagnosis of prostate cancer |
| eventdate_td | Date of consultation |
| pca_symptom | Symptom of possible prostate cancer coded in the consultation |
| psa_test | PSA test performed in the consultation |

Table S3.2 – Variable descriptions

*Statistical analysis*

Summary descriptive statistics of patient demographics were performed. Number of consultations by individual patients for symptoms of possible prostate cancer and/or PSA testing in the 12-months prior to diagnosis were measured and summarised. The diagnostic interval was calculated from the date of first presentation with a symptom that might relate to a prostate cancer to the date of diagnosis in CPRD and summarised with median and interquartile range (IQR). Median diagnostic interval by number of pre-referral consultations was calculated and stratified by type of initial presentation (symptomatic vs opportunistic screening). Primary care interval (date of first presentation to date of first suspected cancer referral) could not be calculated owing to significant levels of missing data on referral dates.

Estimation of the probability of returning to the GP having not been referred following the initial two consultations (one to present with symptoms and a follow-up consultation to discuss PSA results and possible referral) were estimated using the *proportion* command in Stata. A variable was generated that counted the number of consultations for each patient and then estimate the proportion of patients who had consulted three or more times. Stratification by year of diagnosis was undertaken, as were analyses to compare outcomes in three time periods relating to publication of NICE guidelines on cancer diagnosis in primary care (pre-25/06/2005 vs 26/06/2005–22/06/2015 vs 23/06/2015–31/12/2017). Chi-squared testing was performed for changes between the NICE guideline time periods. Sub-group analyses were performed for different initial presentation types.

**Results**

78,812 consultations for 41,115 patients in this CPRD dataset were analysed. The mean number of pre-diagnostic consultations per patient was 1.9 (SD 0.8). Of the 34,410 initial consultations during the study period, 23,201 (67.4%) were for an initial PSA test without any recorded symptoms and classified as opportunistic screening. LUTS was the next most common initial presenting complaint (7,467 / 34,410 [21.7%]). See Table S4.3 for further details regarding clinical coding associated with initial consultations.

| **Coded problem** | **n (%)** |
| --- | --- |
| LUTS | 7,467 (21.7%) |
| Haematuria | 1,352 (3.9%) |
| Erectile dysfunction | 1,175 (3.4%) |
| Abnormal DRE | 361 (1.0%) |
| Opportunistic PSA test | 23,201 (67.4%) |
| Missing | 854 (2.5%) |

Table S3.3 – Proportions of index presentation types for patients with prostate cancer

n – number of patients; % - proportion; DRE – Digital Rectal Examination; PSA – Prostate Specific Antigen

49,619 PSA tests were performed on the patients in this cohort. From the initial PSA tests undertaken on patients who had at least one test taken (n = 34,410), the median PSA result was 10.8ng/mL (IQR 6.3, 27.1). Initial PSA results were higher for patients with symptoms compared to those undergoing opportunistic screening (median PSA 14.6ng/mL [IQR 7, 43.6] vs 11.1ng/mL [IQR 6.4, 28.7]). Further details about PSA test results can be found in Table S4.4.

| **Overall (n = 34,410)** | |
| --- | --- |
|  | **Median (IQR)** |
| PSA level (ng/mL) | 10.8 (6.3, 27.1) |
|  | **n (%)** |
| PSA above lab reference range | 13,446 (39.1%) |
| PSA above NICE threshold | 21,544 (62.6%) |
| PSA > 3ng/mL | 22,132 (64.3%) |
| **Symptomatic** | |
|  | **Median (IQR)** |
| PSA level (ng/mL) | 14.6 (7, 43.6) |
|  | **n (%)** |
| PSA above lab reference range | 4,326 (59.3%) |
| PSA above NICE threshold | 6,678 (91.6%) |
| PSA > 3ng/mL | 6,866 (94.1%) |
| **Opportunistic screening** | |
|  | **Median (IQR)** |
| PSA level (ng/mL) | 11.1 (6.4, 28.7) |
|  | **n (%)** |
| PSA above lab reference range | 13,446 (56.6%) |
| PSA above NICE threshold | 21,544 (90.7%) |
| PSA > 3ng/mL | 22,132 (93.2%) |

Table S3.4 – Initial PSA results for patients with prostate cancer

n – number of patients; % - proportion; PSA – Prostate Specific Antigen; ng/mL – nanograms per millilitre; NICE – National Institute for health and Care Excellence; IQR – Interquartile Range

The median diagnostic interval for the cohort was 88 days (IQR 25, 197), with minimal differences between symptomatic patients (91 days IQR 45, 189) and patients undergoing opportunistic screening (87 days IQR 40, 203). Median diagnostic interval was much longer before the introduction of NICE guidance compared to afterwards (107 days IQR 52,217 vs 83 IQR 44, 161). The median diagnostic interval increased for each additional pre-referral consultation (See Table S4.5). 29.0% (9,994 / 34,410) of the cohort had three or more primary care consultations in the year prior to prostate cancer diagnosis, and this proportion was higher for symptomatic patients than patients initially presenting for opportunistic screening throughout the study period (See Table S4.6). Bootstrapping techniques using 1,000 repetitions estimated the proportion of patients with symptoms at initial presentation having three or more pre-referral consultations in primary care to be 13.82% (95% CI 13.53, 14.12).

|  | **1 consult** | **2 consults** | **3 consults** | **4 consults** | **5+ consults** |
| --- | --- | --- | --- | --- | --- |
| **Overall** | | | | | |
| n (%) | 13,072 | 11,343 | 6,062 | 2,494 | 571 |
| DI (median, IQR) | 54 (27, 107) | 91 (45, 191) | 139 (66, 255) | 203 (101, 296) | 274 (195, 337) |
| **Symptomatic** | | | | | |
| n (%) | 1,932 | 3,665 | 2,734 | 1,248 | 320 |
| DI (median, IQR) | 66 (30, 144) | 73 (41, 144) | 94 (49, 187) | 140 (76, 238) | 240 (162, 316) |
| **Opportunistic screening** | | | | | |
| n (%) | 12,536 | 6,985 | 2,543 | 762 | 136 |
| DI (median, IQR) | 55 (27, 109) | 110 (51, 220) | 209 (98, 293) | 273 (184, 327) | 314 (231, 347) |

Table S3.5 – Diagnostic interval (DI) for patients by number of pre-referral consultations

n – number of patients; % - proportion; DI – Diagnostic interval; IQR – Interquartile range

|  | **Whole cohort**  **n = 34,410** | **Pre-CG27**  **n = 7,799** | **CG27**  **n = 23,644** | **NG12**  **n = 2,966** |
| --- | --- | --- | --- | --- |
| All patients* | 9,994 (29.0%) | 1,941 (24.9%) | 7,190 (30.4%) | 863 (29.1%) |
| Symptomatic patients* | 4,757 (13.8%) | 996 (12.8%) | 3,362 (14.2%) | 399 (13.5%) |
| Opportunistic screening* | 5,184 (15.1%) | 931 (11.9%) | 3,793 (16.0%) | 460 (15.5%) |

Table S3.6 – Proportion of patients with 3+ pre-referral consultations overall, and within time periods relating to NICE suspected cancer guidelines.

* p<0.001 for difference between NICE time periods

CG27 – Clinical guideline 27; NG12 – NICE guideline 12

**Discussion**

This secondary analysis of a primary care dataset of men with prostate cancer showed that 29.0% of patients had three or more pre-referral primary care consultations overall, with patients having symptoms at initial presentation much more likely to have three or more pre-referral consultations. Median diagnostic interval increased with an increasing number of pre-referral consultations. Initial PSA levels were higher in patients presenting with symptoms compared to those undergoing initial opportunistic screening.

Estimates of the proportion of symptomatic patients with three or more consultations in this CPRD dataset were consistent with other studies. Lyratzopoulos *et al* found (2013) 15.2% of prostate cancer patients in the 2009-2010 National Cancer Diagnosis Audit (NCDA) had three or more pre-referral consultations. However, the median diagnostic interval for symptomatic prostate cancer patients in this analysis (91 days) was significantly higher than reported in the analysis of a more recent round of the NCDA in 2014 (55.5 days). This appears to be at least in part due to the long time period covered by the IMPACT study data set (1999 – 2017), but even in recent years the diagnostic interval was much higher.

The strengths of this analysis lie in the large numbers of patients, covering a significant time period, with relatively complete primary care consultation data. Some patients may have been misclassified as undergoing opportunistic screening due to the 12-month pre-diagnosis window of the data and a possible lack of coding of symptoms in the GP record. PSA data is also skewed by only assessing patients with a diagnosis of prostate cancer.
