# Supplementary material for: Early economic evaluation of magnetic resonance imaging for prostate cancer detection in primary care
Source: BJUI Compass. 2024 Jul 10;5(9):855–64. doi: 10.1002/bco2.409 (PMC11420105; doi:10.1002/bco2.409)
Supplement: Supplementary file 4 — Figures S4.1 & S4.2. Tornado plots for deterministic sensitivity analyses in screening patients Figures S4.3 & S4.4. Tornado plots for deterministic sensitivity analyses in symptomatic patients [file BCO2-5-855-s004.docx]

Supplementary file 4

Figures S4.1 & S4.2 – Tornado plots for deterministic sensitivity analyses in screening patients

Figures S4.3 & S4.4 – Tornado plots for deterministic sensitivity analyses in symptomatic patients
