# Supplementary material for: Early economic evaluation of magnetic resonance imaging for prostate cancer detection in primary care
Source: BJUI Compass. 2024 Jul 10;5(9):855–64. doi: 10.1002/bco2.409 (PMC11420105; doi:10.1002/bco2.409)
Supplement: Supplementary file 5 — Table S5. Probabilistic sensitivity analyses of estimated costs and utilities associated with the three strategies in the model [file BCO2-5-855-s002.docx]

Supplementary file 5

| **Option** | **Costs** | **Annual utility** | **Incremental costs (relative to PSA)** | **Incremental utility** | **ICER** |
| --- | --- | --- | --- | --- | --- |
| PSA (mean [95% credible interval)] – Symptomatic patients | | | | | |
| PSA pathway | £1,302.73 (£1,204.67, £1,400.78) | 0.9894575 (0.9889880, 0.9899271) |  |  |  |
| mpMRI pathway | £930.46 (£872.32, £988.60) | 0.9894328 (0.9890866, 0.9897791) | -£372.26 | -2.47031 x 10^-5^ | £15,069,451.36 |
| bpMRI pathway | £610.66 (£582.74, £638.59) | 0.9891136 (0.9887327, 0.9894946) | -£692.06 | -0.0003439 | £2,012,443.21 |
| PSA (mean [95% credible interval)] – Screening patients | | | | | |
| PSA pathway | £735.27 (£683.77, £786.77) | 0.9899513 (0.9895040, 0.9903987) |  |  |  |
| mpMRI pathway | £531.53 (£499.01, £564.06) | 0.9897120 (0.9893488, 0.9900753) | -£203.74 | -0.0002392 | £851,434.81 |
| bpMRI pathway | £308.38 (£292.90, £323.86) | 0.9894773 (0.9890787, 0.9898759) | -£426.89 | -0.0004740 | £900,600.60 |

Table S5 – Probabilistic sensitivity analyses of estimated costs and utilities associated with the three strategies in the model

PSA – Prostate Specific Antigen; mpMRI – Multiparametric Magnetic Resonance Imaging; bpMRI – Biparametric Magnetic Resonance Imaging; ICER – Incremental Cost Effectiveness Ratio
