# Supplementary material for: Early economic evaluation of magnetic resonance imaging for prostate cancer detection in primary care
Source: BJUI Compass. 2024 Jul 10;5(9):855–64. doi: 10.1002/bco2.409 (PMC11420105; doi:10.1002/bco2.409)
Supplement: Supplementary file 6 — Figure S6.1 & S6.2. Cost‐effectiveness scatter plots for screening patients Figure S6.3 & S6.4. Cost‐effectiveness scatter plots for symptomatic patients [file BCO2-5-855-s001.docx]

Supplementary file 6

Figure S6.1 & S6.2 – Cost-effectiveness scatter plots for screening patients

Figure S6.3 & S6.4 – Cost-effectiveness scatter plots for symptomatic patients
