# Supplementary material for: Early economic evaluation of magnetic resonance imaging for prostate cancer detection in primary care
Source: BJUI Compass. 2024 Jul 10;5(9):855–64. doi: 10.1002/bco2.409 (PMC11420105; doi:10.1002/bco2.409)
Supplement: Supplementary file 7 — Figure S7.1 & S7.2. Cost‐effectiveness acceptability curves for screening patients Figure S7.3 & S7.4. Cost‐effectiveness acceptability curves for symptomatic patients [file BCO2-5-855-s005.docx]

Supplementary file 7

Figure S7.1 & S7.2 – Cost-effectiveness acceptability curves for screening patients

Figure S7.3 & S7.4 – Cost-effectiveness acceptability curves for symptomatic patients
